# Complete Circular Genome Sequence of a Multidrug-Resistant Escherichia coli Strain from Cuba Obtained with Nanopore and Illumina Hybrid Assembly

**DOI:** 10.1128/MRA.01269-19

**Published:** 2019-11-27

**Authors:** Rosa Elena Hernández-Fillor, Michael Brilhante, Ivette Espinosa, Vincent Perreten

**Affiliations:** a Institute of Veterinary Bacteriology, Vetsuisse Faculty, University of Bern, Bern, Switzerland; b National Centre for Animal and Plant Health (CENSA), San José de las Lajas, Mayabeque, Cuba; c Graduate School of Cellular and Biomedical Sciences, University of Bern, Bern, Switzerland; University of Maryland School of Medicine

## Abstract

The complete genome sequence of a multidrug-resistant Escherichia coli strain isolated from a healthy pig in Cuba was determined using short and long reads. This strain carried four plasmids, including a 42,683-kb IncX1 plasmid, which contains the third-generation cephalosporin resistance gene *bla*_CTX-M-32_ together with other disinfectant and antibiotic resistance genes.

## ANNOUNCEMENT

Commensal Escherichia coli from animals represents a reservoir of acquired antibiotic resistance elements, which can be transferred to pathogenic E. coli strains (1). The multidrug-resistant (MDR) E. coli strain described here is part of a larger study screening pigs for the presence of third-generation cephalosporin-resistant E. coli in Cuba, where *bla*_CTX-M-32_ was found to be a predominant third-generation cephalosporin resistance gene. We completely sequenced one of these CTX-M-32-containing E. coli strains with both short and long reads to gain information on the structure of the mobile genetic elements and localization of the antibiotic resistance genes.

A rectal swab from a healthy pig was collected in a swine facility of the Matanzas municipality in July 2016 and was grown overnight at 37°C on a MacConkey (BioCen, Cuba) agar plate supplemented with cefotaxime (4 μg/ml) (Sigma-Aldrich, USA). Identification of the E. coli (strain PK6) isolate was performed by matrix-assisted laser desorption ionization–time of flight (MALDI-TOF) mass spectrometry (Microflex LT, Germany). Genomic DNA was extracted from an overnight culture in agitated LB broth at 37°C using the DNeasy blood and tissue extraction kit (Qiagen, Germany) and purified using the AMPure XP kit (Beckman Coulter, USA). DNA was quantified using a Qubit 3.0 fluorometer (Invitrogen, USA), and 1.5 μg of DNA was used for sequencing.

Whole-genome sequencing was performed with both long and short reads to obtain a complete scaffold of the genome and an accurate sequence. Short reads were obtained using the NovaSeq 6000 S2 reagent kit (300 cycles) and an S2 flow cell on a NovaSeq 6000 (2 × 150-bp paired-end reads) system (Illumina, USA) at Eurofins Genomics GmbH, Germany, yielding 6,411,400 reads (1.9 Gbp of data and 385× coverage). Default parameters were used for all the following bioinformatic software. In order to remove adaptors and low-quality reads (quality value [QV], ≤20), short reads were trimmed using Trimmomatic v0.36 (illuminaclip:TruSeq3-PE.fa:2:30:10, LEADING:3, TRAILING:3, SLIDINGWINDOW:4:15, and MINLEN:36) (2). For long-read sequencing, the genomic DNA was sheared using Covaris g-TUBES to generate fragments of around 20 kb, and the resulting total DNA was used. The library was prepared using the ligation sequencing kit 1D SQK-LSK108 and native barcoding kit 1D EXP-NBD103 in an R9.4 SpotON flow cell with a MinION Mk1B device from Oxford Nanopore Technologies (ONT; United Kingdom), yielding 269,705 reads (1.5 Gbp of data, 309× coverage, mean read length of 6,664 bp, and *N*_50_ of 11,844 bp). The reads were base called and demultiplexed using Guppy basecaller (v2.3.7) and Guppy barcoder (v2.3.7), respectively (ONT). Hybrid *de novo* assembly of both short and long reads and circularization of the replicons were performed using the Unicycler v0.4.4 pipeline (3). The genome of E. coli PK6 was annotated using the NCBI Prokaryotic Genome Annotation Pipeline (4). The complete genome consisted of five circular contigs that are described in Table 1, with a sum of 4,789,128 bp and a G+C content of 50.8%, 4,582 coding sequences, 89 tRNAs, 22 rRNAs, 1 transfer-messenger RNA (tmRNA), and 10 noncoding RNAs (ncRNAs).

**TABLE 1:** Characteristics of the genome of E. coli strain PK6 from Cuba

Genetic element	Sequence name	Assembly size (bp)	G+C content (%)	Multiplicity (*k*)^*a*^	Incompatibility group (plasmids)^*b*^	Antimicrobial resistance mechanisms^*c*^
Chromosome	PK6CUB-RH_CHR	4,726,526	50.8	1.00	NA	QnrB19, TEM-1B, Tet(B), GyrA (S83L), ParC (S80I)
Plasmid 1	pRHEcCUB-1	42,683	48.5	1.30	IncX1	AadA1, AadA2, CmlA1, DfrA12, QacH2, Sul3 (In*640*); CTX-M-32
Plasmid 2	pRHEcCUB-2	12,602	45.5	2.94	NI	
Plasmid 3	pRHEcCUB-3	4,593	50.4	13.53	NI	
Plasmid 4	pRHEcCUB-4	2,724	47.7	4.14	NI	

a Multiplicity was calculated using Unicylcler to distinguish between the single-copy contig (*k *= 1) and the multiple contigs (*k *> 1) (3).

b NA, not applicable; NI, not identified using PlasmidFinder (v2.0) (6).

c Antimicrobial resistance mechanisms were AadA1 and AadA2, streptomycin/spectinomycin adenylyltransferases; CmlA1, chloramphenicol efflux protein; CTX-M-32, extended-spectrum β-lactamase for cephalosporin resistance; DfrA12, trimethoprim-resistant dihydrofolate reductase; GyrA (S83L) and ParC(S80I), serine-to-leucine substitution at position 83 in topoisomerase II (GyrA) and serine-to-isoleucine substitution at position 80 in topoisomerase IV (ParC) for high-level resistance to fluoroquinolones; QacH2, quaternary ammonium compound exporter protein; QnrB19, DNA gyrase protection protein for low-level resistance to fluoroquinolones; Sul3, sulfonamide-resistant dihydropteroate synthase; TEM-1B, β-lactamase; Tet(B), tetracycline efflux protein; and In*640*, integron containing the *qacH2*, *aadA1*, *aadA2*, *cmlA1*, and *dfrA12* genes as determined using the INTEGRALL database.

Strain PK6 belongs to sequence type 1695 (ST1695), as determined by *in silico* analysis using MLST (v2.0) based on the Achtman scheme for E. coli (5). Only one plasmid, namely, pRHEcCUB-1, could be assigned to an incompatibility group (IncX1) using PlasmidFinder (v2.0) (6).

Antibiotic resistance genes were detected *in silico* with ResFinder (v3.1) (7) on the chromosome as well as on the 42,683-bp IncX1 plasmid pRHEcCUB-1. Plasmid pRHEcCUB-1 also contains a gene for resistance to a quaternary ammonium compound disinfectant (Table 1).

This report provides information on the complete and circularized genome of an MDR and extended-spectrum β-lactamase-producing E. coli isolate from Cuba. The sequence can serve as a baseline for future molecular epidemiological studies and for surveillance of antibiotic resistance in bacteria from humans and animals in Cuba.

### Data availability.

The complete chromosome and plasmid sequences of E. coli PK6 have been deposited into GenBank under accession numbers CP042588, CP042589, CP042590, CP042591, and CP042592. The associated BioProject and BioSample accession numbers are PRJNA559061 and SAMN12512712, respectively. The raw reads were deposited into the SRA database with accession numbers SRR10015223 (Illumina) and SRR10015224 (ONT).
